# Cutaneous absorption of Oleander: Fact or fiction

**DOI:** 10.4103/0974-2700.44682

**Published:** 2009

**Authors:** S Senthilkumaran, S Saravanakumar, P Thirumalaikolundusubramanian

**Affiliations:** Vinayaka Mission University Hospitals, Salem, TamilNadu, India

**Keywords:** Oleander leaf extract, reversible cardiac conduction disorder, transcutaneous absorption

## Abstract

Cardiac conduction disorders following oral ingestion of Oleander plant materials were documented earlier. Transcutaneous absorption of yellow oleander (Thevetia peruviana) leaf extract applied over non intact skin (raw wound) resulting in reversible cardiac conduction disorder observed in four healthy males who were free from any other systemic or electrolyte or metabolic disorders or exposure to pesticide or toxins is reported for the first time. Their hematological, biochemical, clinical, and echocardiogram status were within normal limits and free of any abnormalities. One among the four, presented for weakness and breathlessness (class II). He had bradycardia with Mobitz II block and hypotension without any other demonstrable localizing signs. The other three were identified in the community and without any symptoms. However, their ECG revealed bradycardia with Mobitz I block in two and complete heart block in the other. All of the four recovered well without any untoward events. Hence, it is suggested that physicians and practitioners have to elicit history and route of administration of unconventional therapy, whenever they are confronted with clinical challenges and during medical emergencies before embarking final decision.

## INTRODUCTION

Unconventional therapies are difficult to define, because they encompass a broad spectrum of practices and beliefs, and the extent of their use and the costs are not known. Oleander plant is a shrub belonging to the family Apocynaceae, and thrives in tropical and subtropical regions. Its materials have cardiotonic properties and hence are used in indigenous medical system. Their poisonous effect and toxicity have been documented earlier.[1] The physiological action of oleander cardenolides is similar to that of classic digitalis glycosides i.e., inhibition of Na+ K+ ATPase. Here we report a series of cases that had conduction disorders due to oleander toxicity through an unusual route (transcutaneous) of entry.

## CASE 1

A 33-year-old male, working in a grocery store presented to emergency room with a history of generalized weakness and breathlessness of class II over a period of two days. There was no history of fever, chest pain, palpitation, syncope or any known allergic disorders. He was a teetotaler and was not under any medications. His medical history otherwise was not significant. On examination, he was found to be conscious, well oriented, moderately built, and well nourished. There was no pallor, pedal edema or any clinical evidence of hypo or hyperthyroidism. He had a pulse rate of 48/min (irregular), BP: 80/60mm of Hg, and respiratory rate of 13/min. He was afebrile and his oxygen saturation on room air was 98%. Auscultation revealed normal heart sounds, no murmur or gallop but his heartbeat was regularly irregular. The lungs were clear. His abdomen and central nervous examinations were normal.

A provisional diagnosis of fatigue for evaluation was made. His capillary blood glucose level was 146 mg/dl and ECG showed bradycardia with Mobitz II block. Since there was symptomatic bradycardia, a trial of 2 mg of atropine was given intravenously and there was no response. He was started on dopamine 5 microgram/kg/min titrated upwards to 10 microgram/kg/min. He was admitted to the intensive care unit for further management. His hematological and biochemical values including thyroid profile, calcium, magnesium, and electrolytes were within normal limits. His Troponin-T, RPR [Rapid Plasma Reagin] test, and HIV status were negative. As he had isolated electrocardiographic features of Mobitz type II block without any demonstrable cause, he was subjected for echocardiogram, which was normal. The cardiologist opined for urgent pacing, but the patient refused. However, with the consent of the patient, his right internal jugular vein was canulated with 14G venflon so as to facilitate emergency transvenous insertion of pacemaker.

He was managed with oral orciprenaline and atropine (sos). Dopamine was slowly tapered and stopped within 36 hours. As the patient was symptomatically improving, a diagnosis of idiopathic second-degree type II A V block was considered. While on re-evaluation of the case, a non-specific penile ulcer was noticed. On careful interrogation, the patient admitted of suffering from penile ulcer for which he applied a native medicine paste on alternative days for a week as advised by a native medical practitioner of his village. The last application of the medication was 24 hours before presenting to our emergency room. He denied any extramarital relationship. Hence, the native medical practitioner was contacted in person to know more about the paste.

On interaction with the native physician concerned, he explained that the paste was made of bark and leaves of yellow oleander plant, tulsi, tamarind, onion, and neem. He also admitted that he was treating all open wounds or ulcers presented to him with the same paste! With the benefit of doubt, the patient was started with multidose activated charcoal (MDAC) for a day. So, the final diagnosis at the time of discharge was drug induced A V block probably due to transcutaneous absorption of oleander. Our diagnosis was supported by normalization of rhythm within 2 days in the absence of offending drugs.

As this has raised the suspicion of transcutaneous absorption of naturally occurring cardiac glycoside (oleander), it was decided to contact the native practitioner and find out if any of his cutaneous ulcer cases were on treatment with similar paste at present. Three more cases with cutaneous ulcer on treatment with similar paste were evaluated at the practitioner's clinic, after obtaining informed consent as all of them refused hospitalization and modern medicines. They were followed up at their residence.

## CASE 2

A 35-year-old well-built male, manual worker was applying similar paste for his (nonspecific) perianal ulcer (5 to 6 mm) following pruritus ani on alternative days for seven days. He did not suffer from any fever, chest pain, palpitation, syncope, breathing difficulties, bleeding episodes, altered bowel habits or urinary disorder. Clinical examination did not reveal any abnormality but for bradycardia. His pulse, BP, respiration, temperature, and oxygen saturation on room air were 46/min (irregular), 100/60 mm of Hg, 15/min, 37°C (oral) and 98%, respectively. There were no overt evidences of cholinergic signs. Auscultation revealed normal heart sounds, no murmur or gallop. Examination of chest, abdomen, and central nervous examination was unremarkable. His ECG showed evidences of complete heart block. His urinalysis, hematological profile, blood chemistry (including thyroid profile, calcium, magnesium, and electrolytes) and echocardiogram were within normal limits. His Troponin-T and HIV status were negative.

## CASE 3

A 30-year-old male farmer applied the same indigenous medicine as liniment on alternative days over the past two weeks for his ulcers (two and size 4 to 5 mm each) in the inguinal region following itching over tinea cruris, as advised by the same native medical practitioner. There were no overt evidences of cholinergic signs. He was asymptomatic; his clinical examination did not reveal any obvious findings. His vitals were stable except for the pulse, which was 45/min (irregular). His ECG showed bradycardia with Mobitz I block. His investigations including serum calcium, magnesium, and electrolytes were within normal limits.

## CASE 4

A 40-year-old male farmer was suffering from nonspecific scrotal ulcer, for which he applied the native medicine locally on alternative days for two weeks. He was apparently normal with stable vitals expect for the pulse which was 45/min (irregular). His ECG showed bradycardia with Mobitz I block. His blood chemistry including calcium, magnesium, and electrolytes were within normal limits.

## DISCUSSION

Unconventional therapies for diseases of private parts are prevalent in the rural areas. Users of unconventional therapy are more likely to seek a traditional healer rather than to consult a qualified doctor. Oleander seeds contain highly toxic cardiac glycosides, including thevetin A, thevetin B, neriifolin, and peruvoside.[2]

In these four cases, the major portion of the paste contained yellow oleander leaves and barks (Thevetia peruviana). On personal enquiry, it was noted that many traditional healers believe that seeds are only toxic and other parts of the plants are nontoxic when applied externally. Interestingly, Ojio and colleagues[3] have identified many cardenolides, which are highly toxic cardiac glycosides in bark, leaves, kernel of the seed, or sap of yellow oleander. The factors responsible for transcutaneous absorption and toxicity in the present cases are likely to be related to patient – area and nature of ulcer, cutaneous circulation, sweating, level of activity, temperature and ability to absorb; plant – part of the plant used, substances/adjuvant mixed with plant material, and freshness of the paste; and prescriber – frequency and duration of application. A combination of them could explain the variation in the ECG changes noticed. The limitation of this case series was lack of laboratory evidence for cardio active steroid due to technical constraints. As cardiac toxicity due to tulsi, tamarind, onion, and neem is almost unknown, it is likely that the extract of the oleander plant materials applied on the raw skin lesions in indigenous medical practice system could have contributed for the cardiac toxicity. Transcutaneous absorption of oleander was not reported earlier. The role of other adjuvants/substances used with oleander might have enhanced the absorption of cardiac glycoside through the raw area needs further exploration.

Clinical and ECG evidences (without cardiac abnormalities) noticed in all the four cases treated with similar preparation (oleander paste) supports our hypotheses of transcutaneous absorption of oleander through non-intact skin ((raw area). Eddelston and colleagues[4] have described variation in the amount of absorption of cardio active toxins from a seed, and the number of seeds ingested did not always correlate with the degree of toxicity, which explains the pattern observed in this series. Case number 2, 3, and 4 were asymptomatic, as they were not involved in active manual work due to treatment. All the four have recovered regular rhythm within a week of withdrawal of offending agent and were stable during a follow up period of one year.

Before the advent of anti-digoxin Fab antitoxin, temporary cardiac pacing was the main stay of treatment for significant digitalis induced dysrhythmias. French retrospective studies[5,6] have compared the outcomes between antitoxin and pacing. They have observed increased complications with temporary pacing than with immunotherapy. Hence, one has to make careful decision before intravenous pacing.

After absorption into the systemic circulation, cardiac glycosides such as digoxin are secreted into the gut lumen by the action of P-glycoprotein.[7,8] The reason for starting MDAC in this patient was to prevent the enterohepatic recycling. In the gut, activated charcoal binds the secreted glycoside and thereby enhances the glycoside excretion and reduces the half life of the cardenolides.[9,10]

It is interesting to note that serum potassium; magnesium and calcium levels were normal in all four patients. The potassium was not elevated in these patients. This might suggest that with transcutaneous absorption, the kinetics is closer to a chronic overdose than an acute oral overdose. Eddelston[11] had reported that a number of patients with severe oleander toxicity had normal or low serum potassium. Therefore, this does not appear to be a reliable marker of toxicity. Moreover, serum potassium may not tally with intracellular potassium.

## CONCLUSION

In view of the above observations, physicians and practitioners have to elicit the history of use of unconventional therapy, whenever they are confronted with clinical challenges and/or medical emergencies. At this juncture, it is worth to explore unconventional therapy and understand these practices, which will improve communication between patients and doctors on one hand and sustain clinical care on the other hand.[12]
